# Dr. Geo. W. Keely

**Published:** 1901-08-15

**Authors:** 


### DR. GEO. W. KEELY.

An address delivered May 9, 1901, by C. M. Wr'ght, A.M., D D.S., before the Alumni Association of the Ohio College of Dental Surgery on the occasion of the unveiling of a tablet to the memory of Dr. G. W. Keely.

Reprinted from *Ohio Journal.*

"If we could sometimes separate ourselves from ourselves—stand aside—and behold our environment, our own town, our intimate friends of the past and the present, as outsiders behold them, we might find it not only interesting but instructive." But this is a difficult thing to do, because of the struggle each of us has in the crowd of daily and hourly events in which we are plunged; because of the hurry and bustle that whirls around us like living panoramas of scenes which we feel bound to notice during every waking hour. Pleasures, worries, griefs, agitate us for the moment and whirl past, and other pleasures, worries and griefs attract our earnest attention; and we have little time for meditative observations of the scenes that have been swept away by the current and only remain with us as memories.

To-day, however, the Alumni of this Ohio College of Dental Surgery will stop for a few moments to erect a tablet to the memory of one of the distinguished members of our society and of our profession, George W. Keely, alumnus, trustee and teacher in his day, and always a warm and earnest friend of this institution.

We will place this side by side with the marble tablets, slight memorials of two confreres, who, with him, have left impresses more enduring than the tablets which we place in the wall of this hall. I refer to Dr. Jas. Taylor and Dr. Geo. Watt.

Taylor, Watt, Keely! Personalities to all older men, names only to others, but names which the history of our beloved profession will ever surround with wreaths of immortelles.

The seeds which these men planted have grown up into mighty trees, with branches spreading out over the entire civilized world. They were among the first "dental teachers," when the number was confined to two or three American colleges. Now the dental teacher can be found in over thirty American colleges, and in nearly every country in the world, European and Oriental. And these were men of such character that we, to-day, are proud to be able to honor them even in this modest way.

George W. Keely was born in Oxford, Ohio, and made that quaint college town with its scholastic atmosphere, its sacred academic groves and cultured traditions, his home all his life ; and died there honored by the entire community.

There he practiced his profession with such distinction and devotion to the welfare of his patients that the township had a feeling of personal ownership that made them proud to point to him as one of the distinguishing features of the place. Then he was so good a man that rich and poor alike looked to him for counsel, comfort and advice in trouble and sickness, and found him the friend indeed.

He seemed in all things the humanist, though. perhaps, too regardless of his own interests. He was so noted a citizen that the bank and business houses of the entire town closed their doors the day of his funeral.

He was of a type far removed from the commercialism that has crept into our profession and shown even by many who sit in high places in the reserved seats of the aristocratic circle.

In all his ways he was the gentleman and the professional man, or, to be more exact, for these terms are synonymous, he was a true professional man, and this implies the other in its highest and broadest meaning.

Dr. Keely sent out a number of students from his office, as was the custom in those days, many of them being under obligation to graduate at this college ; and I will venture to say that each and every one of his students holds the opinion, firmly fixed in his mind, that the influences spread about him during his student days by his friend and preceptor, Dr. Keely, have been potent forces in times of trial and temptation in his professional life ever since. Influences for good, for ethical and high-toned principles toward his professional brethren, and the profession as a profession, a desire to foster honor, faith and charity toward all men! Verily, to be able to exert an influence for good is the highest privilege of duty in man!

Dr. Keely had the artistic temperament and the artistic sense. He was a lover of nature, and a patron and friend of him who showed taste or talent in artistic lines. The young artist looked to Dr. Keely for encouragement, and did not look in vain.

On a knoll, overlooking the valley of a winding stream, in the beautiful little cemetery where the mortal part of our friend lies, and very near to his grave, is an immense boulder with the simple inscription cut into its rough surface, "Charles Barrows, Artist and Soldier, died 1863." To place this huge boulder on the brow of the hill in Dr. Keely's lot must have been a tremendous undertaking with the help attainable in a small town; but Dr. Keely sought the boulder, whose bed was in the creek some miles away, because of an expressed admiration for it by the young artist, Charles Barrows, and with a team of eight horses and what men he could hire, and with his own hands to the work, he succeeded in transferring it to the cemetery and placing it as a monument over his dead artist friend. How much less are we doing for Dr. Keely to-day? This little incident gives a picture of the sentiment and character of George Keely, and it lives in the hearts of all who know about it.

In the profession his especial distinction lay in the correction of the irregularities of the teeth; and the devotion which he gave to his cases, the enthusiasm he displayed in his work was refreshing, and a stimulus to all who came in contact with him.

The valuable "Keely Collection," which was presented to this college by the son of our beloved friend, shows only a part of the great work done by Dr. Keely in this important branch of surgery.

A village dentist! With the birds singing in the boughs just outside his office windows; the public square opposite, with its grass and trees forming a cool and inviting resting place, and adding wonderfully to the attractive beauty of the miniature city. Every tree in the square had been planted under the direction and through the influence of this village dentist, as citizen and councilman.

The farmer's horses, tied in the shade of the square, while the honest yeoman and his good wife, or the blooming country lassies are visiting the shops about the square; the clusters of capped and gowned students sauntering about in the streets! These are the pictures that come to me when I think of Dr. Keely's office. The whole scene being one of peace and rest and quiet; the antithesis of a scene from the window of a metropolitan dentist! But from this village, and this village dentist's surgery radiated an influence that surrounds us to-day, years after the death of the man who spent his life in that quiet spot.

I could enlarge upon the thoughts that I have simply noted, and I could relate incident after incident that came within my knowledge of Dr. Keely during the time of studentship, and years of friendship afterwards; and these incidents would all be of one character, that is, they would show devotion and love of the good he could accomplish in its practice; an unselfishness that was too Tolstoian, possibly, for modern Christianity; an honor sensitive and never failing; a heart true to friend, and kind even to an enemy; a manner open and cheerful and friendly—but, I am trying to separate myself from myself and look on, as an outsider, on this man with this character ; and even as an outsider I must offer to his life and character and memory my sincere admiration.

That this good man's spirit is with us in memory is true, and that it still lives in the unknown—let us accept the reasoning of Cato :

> "It must be so. Plato, thou reasoned well :
> Else whence this pleasing hope, this fond desire,
> This longing after immortality?
> Or, why shrinks the Soul
> Back on herself and startles at destruction?
> 'Tis the divinity that stirs within us.
> 'Tis heaven itself that points out an hereafter
> And intimates Eternity to man."

